# Blocking Wallerian degeneration by loss of Sarm1 does not promote axon resealing in zebrafish

**DOI:** 10.17912/micropub.biology.000283

**Published:** 2020-07-23

**Authors:** Weili Tian, Hernán López-Schier

**Affiliations:** 1 Helmholtz Zentrum Munich; 2 Ingolstaedter landstarsse 1, Munich, Germany

**Figure 1:**
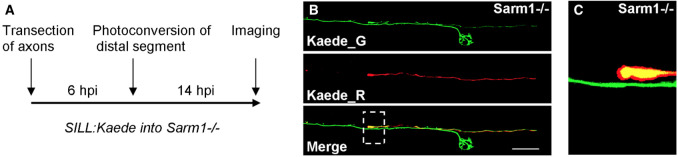
**A.** Schematic representation of the experimental strategy to test resealing of Sarm1-/- transected axons expressing Kaede. Photoconversion of the distal segment was performed 6 hours-post-injury (hpi), and final image taken 14 hpi. **B.** Confocal images of the experiment. Kaede_G is the green (native) form of Kaede in the proximal (regrowing) axon, Kaede_R is the red (photoconverted) form of Kaede in the non-degradable (distal) axon segment. Scale bar is 40μm. The dotted box indicates the site of transection, which shows the juxtaposition between the proximal end of the distal axon segment and the passing regenerated axon. Note the arborization of the green axon below a neuromast. **C.** Zoomed region taken from the dotted box in (B), detailing the interface between the non- degradable distal axon (orange) and the passing regenerated proximal axon (green).

## Description

In *C. elegans* worms, transected axons of mechanosensory neurons reseal to rapidly reconstitute neuronal circuits (Ghosh-Roy, A. *et al.*, 2010; Neumann, B. *et al.*, 2015), which is an effective strategy to recover neural function with high fidelity. Yet, axon resealing has not been observed generally, or specifically in vertebrates. One explanation for this is that degradation of severed axons in vertebrates is too fast to enable resealing. Alternatively, resealing events may have remained hidden owing to the technical difficulties associated to imaging axonal behavior in a living vertebrate. Taking advantage of zebrafish that do not undergo Wallerian axon degeneration due to a mutation in the obligatory pro-degenerative protein Sarm1 (Osterloh JM, *et al.*, 2012; Figley MD, DiAntonio A., 2020; Tian, W., *et al.*, 2020), we tested whether the protracted maintenance of severed axons allows axon-segment resealing. We devised a strategy that employs fluorescent-protein photoconversion to mark individual lateral-line sensory neurons and unambiguously identify proximal and distal axon segments after transection and during regeneration (Figure 1A). To recognize the distal and proximal segments of the same axon after severing and during re-growth, we expressed in individual lateralis neurons the fluorescent protein Kaede, which can be photoconverted with blue light from its native green fluorescence (Kaede_G) to red fluorescence (Kaede_R). Individual Kaede_G-expressing axons in Sarm1 mutants were transected, and 6 hours later the distal segments were illuminated for a short period to render them red fluorescent. Samples were kept in the dark and imaged 14 hours-post-injury to assess resealing. In no instance (N=7) did we see fusion events between the proximal (Kaede_G) and distal (Kaede_R) axon segments (Figure 1B). Proximal axons, instead, grew past the non-degradable distal segments to re-innervate neuromasts (Figure 1C). Whether other neuronal classes undergo axon-segment fusion remains to be tested. Yet, we conclude that axon resealing is not a general feature of the vertebrate nervous system.

## Methods

**Zebrafish strains and husbandry**

Zebrafish (*D. rerio*) were maintained in a centralized facility in accordance to guidelines by the Ethical Committee of Animal Experimentation of the Helmholtz Zentrum München, the German Animal Welfare act Tierschutzgesetz §11, Abs. 1, Nr. 1, Haltungserlaubnis, to European Union animal welfare, and to protocols number Gz.:55.2-1-54-2532-202-2014 and Gz.:55.2-2532.Vet_02-17-187 from the “Regierung von Oberbayern”, Germany. The Sarm1−mutant allele *hzm14* was generated previously using CRISPR/Cas9-mediated genome engineering.

**Laser microsurgery**

To mark lateralis sensory neurons individually, DNA of the SILL:mCherry construct (Pujol-Martí, J., *et al.*, 2012) was injected into eggs of Sarm1−/− zebrafish. Resulting larvae were selected according to red fluorescence in lateralis neurons. Samples were mounted into agarose, and lateral-line peripheral axons were targeted with an ultraviolet laser (350 nm) using the iLasPulse system (Roper Scientific AS, Evry, France), as described previously (Tian, W., *et al.*, 2020). The laser beam was delivered using a 63X water-immersion objective. The laser pulses were calibrated and applied to the target area until a clear gap in the axons was visible. The samples were observed again 1 hour later to confirm complete axon transection.

**Intravital microscopy**

Larval zebrafish were anaesthetized with MS-222 (0.013% M/V) in Danieau’s and mounted in 0.8% low melting-point agarose on 35 mm glass-bottom Petri dishes. Samples were gently pressed against the glass using a hair-loop glued to the tip of a glass pipette, as previously described (Tian, W., *et al.*, 2020). The agarose dome was immersed in Danieau’s with MS-222. Images of cells were acquired using a spinning-disc microscope with a 40X air objective at 28.5 °C. Z-stacks were set to 0.8–1.2 µm intervals. Time intervals were 10 min or 15 min per stack. Representative images were taken when regrowing axons have clearly passed the distal segment or reached the target organ. The resulting raw data were processed, assembled, and analyzed with ImageJ.

## Reagents

Sarm1*^hzm14^* allele; SILL:EGFP; SILL:Kaede DNA constructs.
